# Calorimetric Studies and Thermodynamic Modeling of Ag–Mg–Ti Liquid Alloys

**DOI:** 10.3390/ma17081786

**Published:** 2024-04-12

**Authors:** Weronika Gozdur, Władysław Gąsior, Maciej Zrobek, Andrzej Budziak, Roman Dębski, Wojciech Gierlotka, Magda Pęska, Marek Polański, Adam Dębski

**Affiliations:** 1 Institute of Metallurgy and Materials Science, Polish Academy of Sciences, 25 Reymonta Street, 30-059 Kraków, Poland; w.gozdur@imim.pl (W.G.); w.gasior@imim.pl (W.G.); m.zrobek@imim.pl (M.Z.); 2 Faculty of Energy and Fuels, AGH University of Krakow, Al. Mickiewicza 30, 30-059 Kraków, Poland; budziak@agh.edu.pl; 3 Institute of Computer Science, AGH University of Krakow, Al. Mickiewicza 30, 30-059 Kraków, Poland; rdebski@agh.edu.pl; 4 Department of Materials Science and Engineering, National Dong Hwa University, Hualien 970024, Taiwan; wojtek@gms.ndhu.edu.tw; 5 Department of Functional Materials and Hydrogen Technology, Military University of Technology, 2 Kaliskiego St., 00-908 Warsaw, Poland; magda.peska@wat.edu.pl (M.P.); marek.polanski@wat.edu.pl (M.P.)

**Keywords:** Ag–Mg–Ti, enthalpy of mixing, thermodynamic properties, thermodynamic modeling, calorimetry, liquid alloys, magnesium alloys

## Abstract

Due to the absence of thermodynamic data concerning the Ag–Mg–Ti system in the existing literature, this study aims to fill this gap by offering the outcomes of calorimetric investigations conducted on ternary liquid solutions of these alloys. The measurements were performed using the drop calorimetry method at temperatures of 1294 K and 1297 K for the liquid solutions with the following constant mole fraction ratio: x_Ag_/x_Mg_ = 9/1, 7/3, 1/1, 3/7 [(Ag_0.9_Mg_0.1_)_1−x_Ti_x_, (Ag_0.7_Mg_0.3_)_1−x_Ti_x_, (Ag_0.5_Mg_0.5_)_1−x_Ti_x_, (Ag_0.3_Mg_0.7_)_1−x_Ti_x_)], and x_Ag_/x_Ti_ = 19/1 [(Ag_0.95_Ti_0.05_)_1−x_Mg_x_]. The results show that the mixing enthalpy change is characterized by negative deviations from the ideal solutions and the observed minimal value equals −13.444 kJ/mol for the Ag_0.95_Ti_0.05_ alloy and x_Mg_ = 0.4182. Next, based on the thermodynamic properties of binary systems described by the Redlich–Kister model and the determined experimental data from the calorimetric measurements, the ternary optimized parameters for the Ag–Mg–Ti liquid phase were calculated by the Muggianu model. Homemade software (TerGexHm 1.0) was used to optimize the calorimetric data using the least squares method. Next, the partial and molar thermodynamic functions were calculated and are presented in the tables and figures. Moreover, this work presents, for comparative purposes, the values of the enthalpy of mixing of liquid Ag–Mg–Ti alloys, which were calculated using Toop’s model. It was found that the best agreement between the modeled and experimental data was observed for the cross-sections x_Ag_/x_Ti_ = 19/1 [(Ag_0.95_Ti_0.05_)_1−x_Mg_x_] and x_Ag_/x_Mg_ = 9/1 [(Ag_0.9_Mg_0.1_)_1−x_Ti_x_]. The results of the experiments presented in this paper are the first step in the investigation and future evaluation of the thermodynamics of phases and the calculation of the phase diagram of the silver–magnesium–titanium system.

## 1. Introduction

Magnesium and its alloys find applications across various industries. Due to their notable gravimetric capacity, magnesium and its alloys are considered potential candidates for solid-phase hydrogen storage. Additionally, these alloys exhibit attractive properties such as an excellent strength-to-weight ratio, good fatigue and impact strengths, relatively high thermal and electrical conductivities [1], and excellent biocompatibility. Consequently, they are widely utilized in the medical, aerospace, and automotive sectors. While magnesium-based alloys are extensively researched for various properties in industry, they are not always thoroughly studied in terms of their thermodynamic properties. While the literature on the thermodynamic properties of binary alloys is readily available, the situation changes rapidly when considering materials from ternary or multicomponent systems. For the current case, understanding the properties of binary systems (Ag–Mg, Mg–Ti, and Ag–Ti) represents the initial step in studying the ternary system.

The first research on the Ag–Mg binary system was carried out as early as 1906 [2]. Over the years, researchers have determined the equilibrium phases for this system, and based on the experimental results from the available literature, a phase diagram for the Ag–Mg system has been created [3]. It was found that there are the five following intermediate phases, i.e., AgMg, Ag_3_Mg, ε, κ, and γ′ [4], two eutectic reactions (L ⇆ Ag + AgMg, and L⇆AgMg_3_ + Mg) and one peritectic reaction (AgMg + L ⇆ AgMg_3_), at temperatures of 1029.15 K, 1032.15 ± 0.4 K, and 765.15 K, respectively [5]. The most recent phase diagrams shown by Dai and Malakhov [6] and Dębski et al. [5] were calculated by the CALPHAD method. Information on some thermodynamic properties is available in the literature cited above. A study on the enthalpy of formation for selected solid alloys with up to 80 at. %Mg was performed by Terlicka et al. [4] and the mixing enthalpy change of liquid alloys was studied by Kawakami [7] at 1323 K and Debski et al. [5] at 991 K, 1141 K, and 1272 K, respectively. The results obtained in both cited works are characterized by negative values across the entire range of concentrations and demonstrate a parabolic shape, with a minimum occurring at x_Mg_ = 0.5.

Many researchers have investigated the Ag–Ti system and one of the first studies showed that the components of the system do not mix in the liquid phase; thus, they do not form intermediate phases [8]. It is now agreed that the Ag–Ti equilibrium system includes three solid solutions, βTi, αTi, and Ag, and the AgTi_2_ and AgTi stoichiometric compounds [9]. Recent studies have also confirmed the following transformations: peritectic (L + βTi→AgTi), peritectoid (AgTi + βTi→AgTi_2_), and eutectoid (βTi→AgTi+ αTi) at temperatures of 1294 ± 4 K, 1212 ± 4 K, and 1126 ± 3 K, respectively [9]. It should be noted that there is also another reaction between Ag and AgTi occurring at 1235 ± 5 K (L→Ag + AgTi). The available literature sources do not agree on its nature. Gierlotka et al. [9], Lim et al. [3], and McQuillian [10] describe it as a peritectic reaction, while Arroyave [11] and Emerenko et al. [12] describe it as a eutectic one. As in the case of the Ag–Mg system, the most recent phase diagrams available were calculated using the CALPHAD method and ab initio calculations [9]. Based on these methods, researchers were able to model selected thermodynamic properties and compare them with available experimental results. In the case of the mixing enthalpy changes of liquid solutions, there are observed discrepancies between experimental and calculated results. The modeled values are positive [3,9,11], whereas those determined experimentally are negative [13]. In the work of Gierlotka et al. [9], the heat capacity was additionally calculated for the intermetallic compounds AgTi and AgTi_2_ (for constant volume).

The Mg–Ti system, as presented by Murray [14] shows very low mutual solubility of the individual components, resulting in the absence of intermetallic compounds. The diagram indicates the presence of three equilibrium solid phases: a low-temperature αTi solid solution, a low-temperature Mg solid solution, and a βTi solid phase. It was observed that the solubility of Ti in a solid (Mg) is higher than in a liquid at the melting point of Mg. Thus, it was shown that one of the transformations occurring is peritectic transformation (L + αTi⇆Mg), taking place at 924 K [14]. Moreover, at a temperature of 1155 K, the allotropic transformation of titanium occurs, which is associated with a change in structure from A3⇆A2. The available literature sources include the approximate thermodynamic properties of the system due to the considerable challenges of experimental studies.

The primary objective of this work, in response to the absence of any thermodynamic data, is to measure the change in enthalpy of mixing of liquid Ag–Mg–Ti solutions across several compositions with a constant *x*_Ag_/*x*_Mg_ concentration ratio and to develop ternary interaction parameters of these alloys. In the later parts of this paper, the methodology followed is described. We characterized the experimental part of the research, which is calorimetric measurement. Then, the methodology of the thermodynamic modeling for ternary alloys with symmetrical and asymmetrical mathematical models was explained. Subsequently, the results of the conducted experiment are shown in tables. The last parts of the paper compare the results obtained from the experiment and the calculations performed in chart form with comments, and the closing segment is the conclusion. Studies carried out within the scope of this work are the first step in determining the thermodynamic properties of the Ag–Mg–Ti system and constructing its phase diagram.

## 2. Materials and Methods

**Figure 1:**
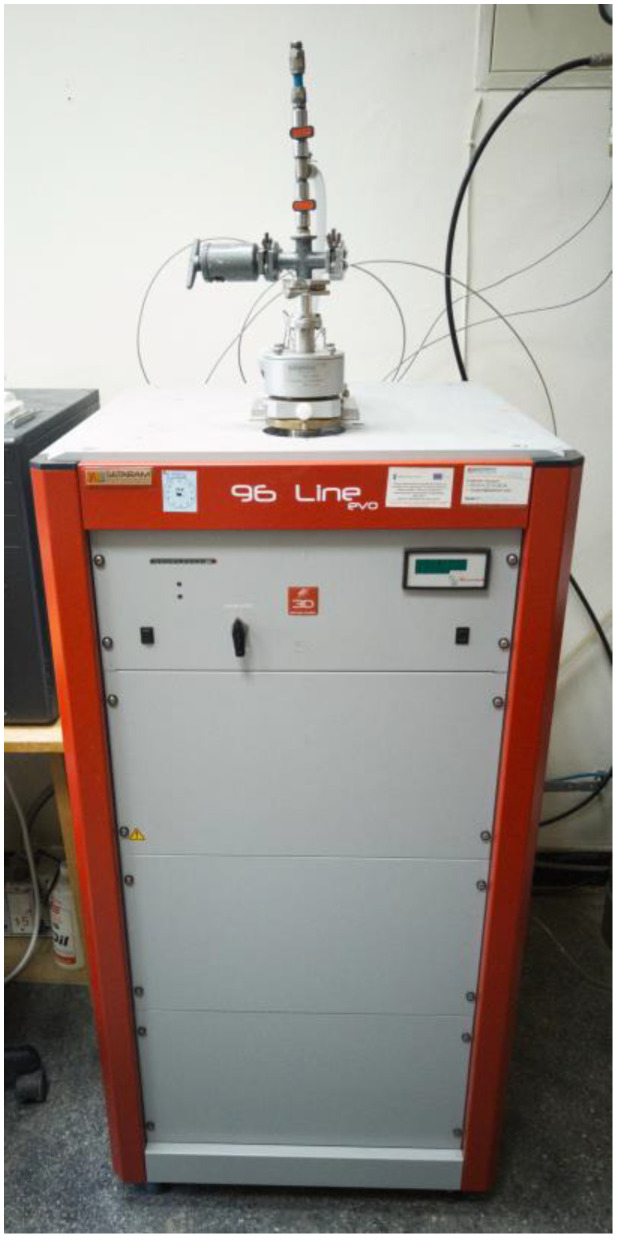
Setaram MHTC 96 Line evo calorimeter.

**Figure 2:**
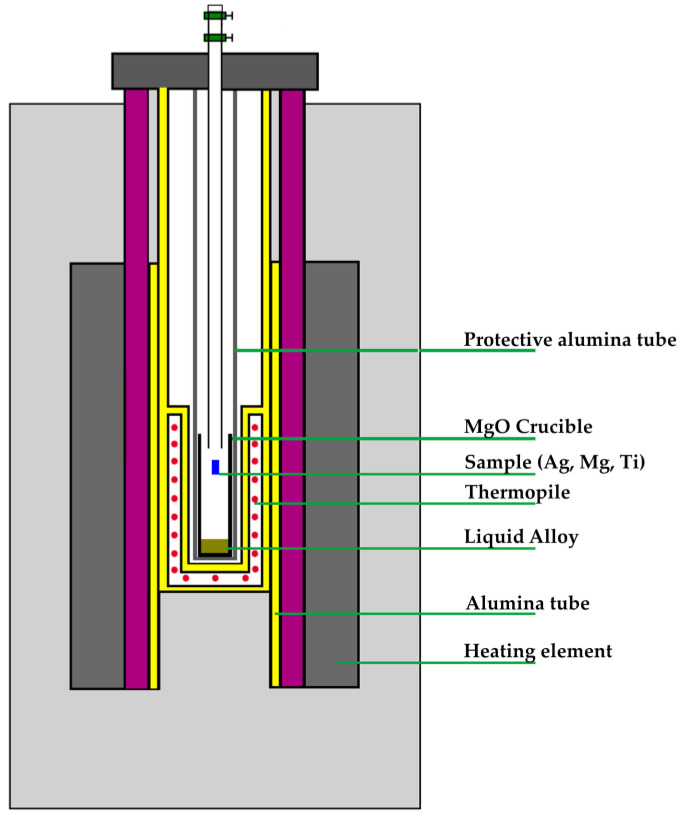
The calorimeter scheme.

The work described in this paper is divided into two phases: one is the experimental measurement and the second is the calculation and modeling. In the first phase, drop calorimetric measurements were conducted in a high-purity argon atmosphere using magnesium oxide (MgO) crucibles (INN-THERM, Trzcianka, Poland) for the metallic bath and the Setaram MHTC 96 Line evo calorimeter (Setaram Instrumentation – KEP technologies, Caluire, France) This calorimeter and a diagram of its internal structure, presented below, represent the principle of the drop method measurement. These are presented in Figure 1 and Figure 2, respectively.

The samples were prepared from the high-purity metal wires specified in Table 1, the shape of which was a cylinder with a diameter of 3 mm and a height of 3 to 20 mm. Before each measurement series, the calorimeter was evacuated several times using a vacuum pump and purged with high-purity argon (Pioniergas, Krakow, Poland). Before being placed into the reaction crucible, the samples were mechanically cleaned with a file to remove any potential surface impurities. After stabilizing the temperature and baseline, the calibration constant was determined using silver samples for all experimental series. The thermal effect was studied six times during the calibration process. Each measurement series consisted of the following stages: the calibration process, measurement of the mixing enthalpy for a starting binary alloy, and measurement of the mixing enthalpy for ternary alloys. These stages could be presented in the form of the following reactions. Starting with the calibration process, *x*Ag_(s, TD)_ → *x*Ag_(l, TM)_, then, measurements of the mixing enthalpies of the binary alloys were conducted. In the case of series A–D, reactions occurred as follows: *x*Ag_(l, TM)_ + *y*Mg_(s, TD)_ → Ag_x_Mg_y(l, TM)_. When the binary starting alloy for the measurements of ternary solutions was Ag–Ti alloy (series E) the reaction occurred as follows: *x*Ag_(l, TM)_ + *z*Ti_(s, TD)_ → Ag_x_Ti_z(l, TM)_. The last stage was measurements of the mixing enthalpies of the ternary alloys; again, between series, reactions had a different course. For a series, A–D occurred according to Ag_x_Mg_y(l, TM)_ + *z*Ti_(s, TD)_ → Ag_x_Mg_y_Ti_z_^i^_(l, TM)_, and for a series E, reactions proceeded as follows: Ag_x_Ti_z(l, TM)_ + *y*Mg_(s, TD)_ → Ag_x_Mg_y_Ti_z_^i^_(l, TM)_. The meanings of the symbols shown are as follows: *T*_D_ is the room temperature (298 K); *T*_M_ is the measurement temperature; “s” is the solid (crystalline); “l” denotes liquid states; x, y, z are the numbers of moles of Ag, Mg, Ti; Ag_x_Mg_y (l, TM)_ or Ag_x_Ti_z(l, TM)_ represent the formation of a starting binary Ag–Mg or Ag–Ti liquid alloy and include the increments in enthalpy for pure magnesium and the melting enthalpy at the measurement temperature; Ag_x_Mg_y_Ti_z_^i^_(l, TM)_ represents the formation of the ith ternary alloy (i = 1, 2, 3, …) and includes the changes in enthalpy for the added metal (Ti or Mg) and the melting enthalpy for the added metal at the measurement temperature.

The parameters of each conducted experiment (e.g., temperatures, argon pressure) are listed in Table 2 and Table 3.

**Figure 3:**
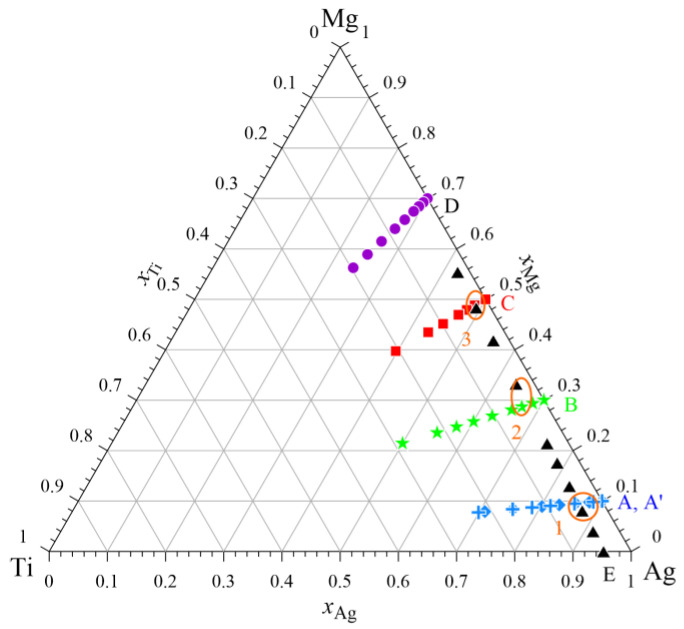
The studied compositions of alloys from the Ag–Mg–Ti system.

The measurements were performed for five separate experimental series for constant ratios of *x*_Ag_/*x*_Mg_ = 9/1, 7/3 1/1, and 3/7, and *x*_Ag_/*x*_Ti_ = 19/1. All alloy compositions for which the mixing enthalpy change was measured in this study along with marked intersection points are shown in Figure 3.

Based on the measured heat effects of the solution of Mg and Ti, the mixing enthalpy change of the Ag–Mg–Ti liquid alloys was calculated by applying the following equations also presented in our earlier work [5]:

(1) $$\Delta_{\mathrm{mix}}H=\frac{\Delta_{\mathrm{mix}}H_{Bin}+\sum H_{\mathrm{DISS}\text{-}X}}{n_{\mathrm{Ag}}+n_{\mathrm{Mg}}+n_{Ti}}$$

(2) $$H_{\mathrm{DISS}\text{-}X}=\left(\Delta H_{\mathrm{Signal}}\cdot K\right)-\left(\Delta H_{X}^{T_{D}\to T_{M}}\cdot n_{X}\right)$$

(3) $$K=\frac{\Delta H_{X}^{T_{D}\to T_{M}}\cdot n_{X}}{\Delta H_{\mathrm{Calibration}}}$$

where $\Delta_{\mathrm{mix}}H_{\mathrm{Bin}}$ is the mixing enthalpy change of the binary alloy; *H*_DISS-X_ is the enthalpy of dissolution of pure magnesium and titanium; *n*_Ag_, *n*_Mg_, *n*_Ti_ are the number of moles of silver, magnesium, and titanium, respectively; ∆*H*_Signal_ is a voltage signal given in μV/s caused by the heat increment that comes from each dropped metal (Mg or Ti); *K* is the calibration constant; $\Delta H_{X}^{T_{D}\to T_{M}}$ is the molar enthalpy difference of the X element (X is magnesium, silver, or titanium) between room temperature (*T*_D_ = 298 K) and the measurement temperature (*T*_M_), calculated using relations in [15]; *n*_X_ (X = Ag, Mg, Ti) is the number of moles of silver, magnesium, and titanium, respectively; and ∆*H*_Calibration_ is a voltage signal given in μV/s caused by the heat increment that comes from the dropped silver sample, which was used for calibration.

In the second phase, the obtained experimental data of the mixing enthalpy change presented in Table 2 and Table 3 were used to calculate the thermodynamic properties (∆_mix_*H*) of liquid Ag–Mg–Ti solutions. For this purpose, two different (symmetrical and asymmetrical) geometrical models were used. The first was a symmetrical Muggianu model [16], with an additional mathematical expression describing the ternary interactions. This model can be expressed as follows:

(4) ΔmixH=∑i∑j>ixi·xj·∑kLi,jLiquidk(xi−xj)k+xixjxk·(L123Liquid0+L123Liquid1·xi+L123Liquid2·xj+L123Liquid3·xk)

The second used model was an asymmetric Toop’s model [17]; this model can be expressed as follows:

(5) $$\Delta_{\mathrm{mix}}H^{\mathrm{Toop}}=\frac{x_{j}}{1-x_{i}}\Delta_{\mathrm{mix}}H_{i,j}\left(x_{i},1-x_{i}\right)+\frac{x_{k}}{1-x_{i}}\Delta_{\mathrm{mix}}H_{i,k}\left(x_{i},{1-x}_{i}\right)+(x_{j}+x_{k}{)}^{2}\Delta_{\mathrm{mix}}H_{j,k}(\frac{x_{j}}{x_{j}+x_{k}},\;\frac{x_{k}}{x_{j}+x_{k}})$$

The parameters in Equations (4) and (5) are as follows: $\Delta_{mix}H$ is the mixing enthalpy change of the liquid Ag–Mg–Ti alloys; $x_{i},\;x_{j},\;x_{k}$ are the Ag, Mg, and Ti mole fractions of the Ag–Mg–Ti alloy, respectively; Li,jLiquidk represents the binary interaction parameters in the Redlich–Kister polynomial [18] for the Ag–Mg, Ag–Ti, and Mg–Ti binary systems; and L123Liquidk (*k* = 0, 1, 2, 3) represents the ternary interaction parameters.

**Figure 4:**
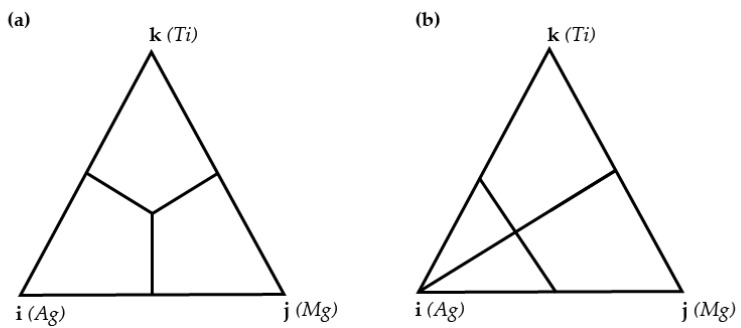
Geometric constructions of thermodynamic properties for ternary systems: (**a**) symmetric—Muggianu model [16], (**b**) asymmetric—Toop model [17].

For the calculations mentioned, we used parameters from binary systems according to the scheme presented in Figure 4.

## 3. Results and Discussion

The obtained values of the integral molar mixing enthalpies, the mole fractions of pure elements, the drop enthalpies, and other important information about the liquid Ag–Mg–Ti alloys studied are listed in Table 2 and Table 3.

**Table 2:** The integral mixing enthalpy of (Ag_0.90_Mg_0.10_)_1−x_T_ix_, (Ag_0.70_Mg_0.30_)_1−x_Ti_x_, (Ag_0.50_Mg_0.50_)_1−x_Ti_x_, and (Ag_0.30_Mg_0.70_)_1−x_Ti_x_ alloys. Standard states: pure liquid metals.

Number of Dropped Moles *n_i_* [mol]	Heat Effect ∆*H*_Signal_ ·*K* [kJ]	Drop Enthalpy *H*_DISS-i_ [kJ]	Mole Fraction *x_i_*	Integral Molar Enthalpy ∆_mix_*H* [kJ/mol]	Partial Molar Enthalpy [kJ/mol]	Standard Uncertainties *u*(∆_mix_*H*) [kJ/mol]
Series A: (Ag_0.90_Mg_0.10_)_1−x_Ti_x_ **Atmosphere**: Argon at pressure *p* = 0.1 MPa. **Starting parameters**: *n*_Ag_ = 0.013123 mol; *K* = 0.000007076 kJ/μVs; *T*_D_ = 298 K; *T*_M_ = 1294 K; $\Delta H_{Ag}^{T_{D}\to T_{M}}$ = 39.7295 kJ/mol; $\Delta H_{Mg}^{T_{D}\to T_{M}}$ = 38.9594 kJ/mol; $\Delta H_{\mathrm{Ti}}^{T_{D}\to T_{M}}$ = 42.9264 kJ/mol. **Standard uncertainties**: *u*(*n*_Ag_) = 0.0000009 mol; *u*(*n*_Mg_) = 0.0000041 mol; *u*(*n*_Ti_) = 0.0000021; *u*(*T*_D_) = 1 K; *u*(*T*_M_) = 1 K; *u*(p) = 10 kPa; *u*(K) = 0.000000151 kJ/μVs.						
***n_Mg_***	**∆*H*_Signal_ ·*K***	***H*_DISS-Mg_**	***x_Mg_***	**∆_mix_H**	**Δ** $\bar{H}$ ***_Mg_***	***u*(∆_mix_*H*)**
0.001461	−0.015129	−0.072	0.1002	−4.939	−49.3	0.025
***n_Ti_***	**∆*H*_Signal_ ·*K***	***H*_DISS-Ti_**	***x_Ti_***	**∆_mix_H**	**Δ** $\bar{H}$ ***_Ti_***	***u*(∆_mix_*H*)**
0.0003384	0.014117	−0.0004	0.0227	−4.855	−1.2	0.05
0.0003928	−0.019389	−0.0362	0.0477	−7.097	−92.3 *	0.08
0.0004930	0.015384	−0.0058	0.0774	−7.241	−11.7 *	0.10
0.0005515	0.018455	−0.0052	0.1085	−7.316	−9.5 *	0.13
0.0010258	0.035827	−0.0082	0.1611	−7.357	−8.0 *	0.18
0.0011239	0.041276	−0.0070	0.2121	−7.287	−6.2 *	0.23
Series A’: (Ag_0.90_Mg_0.10_)_1−x_Ti_x_ **Atmosphere**: Argon at pressure *p* = 0.1 MPa. **Starting parameters**: *n*_Ag_ = 0.013419 mol; *K* = 0.000008991 kJ/μVs; *T*_D_ = 298 K; *T*_M_ = 1297 K; $\Delta H_{Ag}^{T_{D}\to T_{M}}$ = 39.8299 kJ/mol; $\Delta H_{Mg}^{T_{D}\to T_{M}}$ = 39.8299 kJ/mol; $\Delta H_{\mathrm{Ti}}^{T_{D}\to T_{M}}$ = 43.0337 kJ/mol. **Standard uncertainties**: *u*(*n*_Ag_) = 0.0000009 mol; *u*(*n*_Mg_) = 0.0000041 mol; *u*(*n*_Ti_) = 0.0000021; *u*(*T*_D_) = 1 K; *u*(*T*_M_) = 1 K; *u*(p) = 10 kPa; *u*(K) = 0.000000151 kJ/μVs.						
***n_Mg_***	**∆*H*_Signal_ ·*K***	***H*_DISS-Mg_**	***x_Mg_***	**∆_mix_*H***	**Δ** $\bar{H}$ ***_Mg_***	***u*(∆_mix_*H*)**
0.001489	−0.010079	−0.068	0.0999	−4.579	−45.8	0.012
***n_Ti_***	**∆*H*_Signal_ ·*K***	***H*_DISS-Ti_**	***x_Ti_***	**∆_mix_*H***	**Δ** $\bar{H}$ ***_Ti_***	***u*(∆_mix_*H*)**
0.0002402	0.009602	−0.0007	0.0159	−4.555	−3.1	0.02
0.0005369	0.022891	−0.0002	0.0495	−4.412	−0.4	0.05
0.0007584	0.025723	−0.0069	0.0934	−4.629	−9.1	0.08
0.0006246	0.014385	−0.0125	0.1266	−5.192	−20.0	0.09
0.0007145	−0.019600	−0.0503	0.1617	−7.814	−70.5 *	0.11
0.0014164	0.048640	−0.0123	0.2235	−7.879	−8.7 *	0.15
Series B: (Ag_0.70_Mg_0.30_)_1−x_Ti_x_ **Atmosphere**: Argon at pressure *p* = 0.1 MPa. **Starting parameters**: *n*_Ag_ = 0.013119 mol; *K* = 0.000007296 kJ/μVs; *T*_D_ = 298 K; *T*_M_ = 1294 K; $\Delta H_{Ag}^{T_{D}\to T_{M}}$ = 39.7295 kJ/mol; $\Delta H_{Mg}^{T_{D}\to T_{M}}$ = 38.9594 kJ/mol; $\Delta H_{\mathrm{Ti}}^{T_{D}\to T_{M}}$ = 42.9264 kJ/mol. **Standard uncertainties**: *u*(*n*_Ag_) = 0.0000009 mol; *u*(*n*_Mg_) = 0.0000041 mol; *u*(*n*_Ti_) = 0.0000021; *u*(*T*_D_) = 1 K; *u*(*T*_M_) = 1 K; *u*(p) = 10 kPa; *u*(K) = 0.000000030 kJ/μVs.						
***n_Mg_***	**∆*H*_Signal_ ·*K***	***H*_DISS-Mg_**	***x_Mg_***	**∆_mix_*H***	**Δ** $\bar{H}$ ***_Mg_***	***u*(∆_mix_*H*)**
0.005624	−0.003969	−0.223	0.3001	−11.902	−39.7	0.001
***n_Ti_***	**∆*H*_Signal_ ·*K***	***H*_DISS-Ti_**	***x_Ti_***	**∆_mix_*H***	**Δ** $\bar{H}$ ***_Ti_***	***u*(∆_mix_*H*)**
0.0004262	−0.001736	−0.020	0.0222	−12.682	−47.0	0.001
0.0004492	0.006449	−0.013	0.0446	−13.046	−28.6	0.003
0.0004283	0.013037	−0.005	0.0650	−13.034	−12.5	0.005
0.0008816	0.033552	−0.004	0.1044	−12.690	−4.9	0.012
0.0009171	0.042679	0.003	0.1420	−12.006	3.6	0.020
0.0009150	0.039681	0.000	0.1765	−11.506	0.4	0.027
0.0011365	0.060138	0.011	0.2157	−10.483	10.0	0.037
0.0023398	0.080252	−0.020	0.2856	−10.318	−8.6 *	0.049
Series C: (Ag_0.50_Mg_0.50_)_1−x_Ti_x_ **Atmosphere**: Argon at pressure *p* = 0.1 MPa. **Starting parameters**: *n*_Ag_ = 0.010210 mol; *K* = 0.000007166 kJ/μVs; *T*_D_ = 298 K; *T*_M_ = 1298 K; $\Delta H_{Ag}^{T_{D}\to T_{M}}$ = 39.8634 kJ/mol; $\Delta H_{Mg}^{T_{D}\to T_{M}}$ = 39.0967 kJ/mol; $\Delta H_{\mathrm{Ti}}^{T_{D}\to T_{M}}$ = 43.0695 kJ/mol. **Standard uncertainties**: *u*(*n*_Ag_) = 0.0000009 mol; *u*(*n*_Mg_) = 0.0000041 mol; *u*(*n*_Ti_) = 0.0000021; *u*(*T*_D_) = 1 K; *u*(*T*_M_) = 1 K; *u*(p) = 10 kPa; *u*(K) = 0.000000123 kJ/μVs.						
***n_Mg_***	**∆*H*_Signal_ ·*K***	***H*_DISS-Mg_**	***x_Mg_***	**∆_mix_*H***	**Δ** $\bar{H}$ ***_Mg_***	***u*(∆_mix_*H*)**
0.003670	−0.012785	−0.156	0.2644	−11.258	−42.6	0.016
0.003275	0.048014	−0.080	0.4048	−13.774	−24.4	0.064
0.003279	0.088052	−0.040	0.5003	−13.529	−12.2	0.138
***n_Ti_***	**∆*H*_Signal_ ·*K***	***H*_DISS-Ti_**	***x_Ti_***	**∆_mix_*H***	**Δ** $\bar{H}$ ***_Ti_***	***u*(∆_mix_*H*)**
0.0005160	0.019757	−0.0025	0.0246	−13.313	−4.8	0.154
0.0003990	0.019514	0.0023	0.0429	−12.955	5.8	0.169
0.0004304	0.011631	−0.0069	0.0618	−13.016	−16.0	0.179
0.0008649	0.037429	0.0002	0.0976	−12.511	0.2	0.207
0.0008712	0.114159	0.0766	0.1310	−8.789	88.0 *	0.290
0.0022124	0.072838	−0.0224	0.2058	−8.906	−10.1 *	0.339
Series D: (Ag_0.30_Mg_0.70_)_1−x_Ti_x_ **Atmosphere**: Argon at pressure *p* = 0.1 MPa. **Starting parameters**: *n*_Ag_ = 0.010581 mol; *K* = 0.000007126 kJ/μVs; *T*_D_ = 298 K; T_M_ = 1297 K; $\Delta H_{Ag}^{T_{D}\to T_{M}}$ = 39.8299 kJ/mol; $\Delta H_{Mg}^{T_{D}\to T_{M}}$ = 39.0624 kJ/mol; $\Delta H_{\mathrm{Ti}}^{T_{D}\to T_{M}}$ = 43.0337 kJ/mol. **Standard uncertainties**: *u*(*n*_Ag_) = 0.0000009 mol; *u*(*n*_Mg_) = 0.0000041 mol; *u*(*n*_Ti_) = 0.0000021; *u*(*T*_D_) = 1 K; *u*(*T*_M_) = 1 K; *u*(p) = 10 kPa; *u*(K) = 0.000000334 kJ/μVs.						
***n_Mg_***	**∆*H*_Signal_ ·*K***	***H*_DISS-Mg_**	***x_Mg_***	**∆_mix_*H***	**Δ** $\bar{H}$ ***_Mg_***	***u*(∆_mix_*H*)**
0.00822	0.060154	−0.261	0.4373	−13.885	−31.7	0.150
0.00822	0.233755	−0.087	0.6085	−12.894	−10.6	0.555
0.00824	0.281176	−0.041	0.7000	−11.036	−4.9	0.929
***n_Ti_***	**∆*H*_Signal_ ·*K***	***H*_DISS-Ti_**	***x_Ti_***	**∆_mix_*H***	**Δ** $\bar{H}$ ***_Ti_***	***u*(∆_mix_*H*)**
0.0003928	0.008879	−0.0080	0.0110	−11.140	−20.4	0.385
0.0004241	0.011736	−0.0065	0.0226	−11.189	−15.4	0.401
0.0005223	0.017379	−0.0051	0.0366	−11.169	−9.8	0.423
0.0009150	0.033982	−0.0054	0.0601	−11.040	−5.9	0.465
0.0010592	0.037067	−0.0085	0.0859	−10.958	−8.0	0.510
0.0015606	0.054283	−0.0129	0.1214	−10.853	−8.2	0.574
0.0017674	0.067073	−0.0090	0.1585	−10.609	−5.1	0.649
0.0019700	0.062100	−0.0227	0.1963	−10.650	−11.5 *	0.715
0.0019930	0.056292	−0.0295	0.2312	−10.830	−14.8 *	0.773

* liquid–solid alloys.

**Table 3:** The integral mixing enthalpy of (Ag_0.95_Ti_0.05_)_1−x_Mg_x_ alloys. Standard states: pure liquid metals.

Number of Dropped Moles *n_i_* [mol]	Heat Effect ∆*H*_Signal_ ·*K* [kJ]	Drop Enthalpy *H*_DISS-i_ [kJ]	Mole Fraction *x_i_*	Integral Molar Enthalpy ∆*_mix_H* [kJ/mol]	Partial Molar Enthalpy [kJ/mol]	Standard Uncertainties *u*(∆_mix_*H*) [kJ/mol]
Series E: (Ag_0.95_Ti_0.05_)_1−x_Mg_x_ **Atmosphere**: Argon at pressure *p* = 0.1 MPa. **Starting parameters**: *n*_Ag_ = 0.025064 mol; *K* = 0.000006794 kJ/μVs; *T*_D_ = 298 K; *T*_M_ = 1296 K; $\Delta H_{Ag}^{T_{D}\to T_{M}}$ = 39.7964 kJ/mol; $\Delta H_{Mg}^{T_{D}\to T_{M}}$ = 39.0281 kJ/mol; $\Delta H_{\mathrm{Ti}}^{T_{D}\to T_{M}}$ = 42.9979 kJ/mol. **Standard uncertainties**: *u*(*n*_Ag_) = 0.0000009 mol; *u*(*n*_Mg_) = 0.0000041 mol; *u*(*n*_Ti_) = 0.0000021; *u*(*T*_D_) = 1 K; *u*(*T*_M_) = 1 K; *u*(p) = 10 kPa; *u*(K) = 0.000000158 kJ/μVs.						
***n_Ti_***	**∆*H*_Signal_ ·*K***	***H*_DISS-Mg_**	***x_Ti_***	**∆_mix_*H***	**Δ** $\bar{H}$ ***_Ti_***	***u*(∆_mix_*H*)**
0.00125	0.020789	−0.033	0.0475	−1.251	−26.4	0.018
***n_Mg_***	**∆*H*_Signal_ ·*K***	***H*_DISS-Mg_**	***x_Mg_***	**∆_mix_*H***	**Δ** $\bar{H}$ ***_Mg_***	***u*(∆_mix_*H*)**
0.00109	−0.009531	−0.0519	0.0396	−3.097	−47.8	0.026
0.00123	−0.005510	−0.0534	0.0808	−4.828	−43.5	0.031
0.00160	−0.010782	−0.0731	0.1293	−6.991	−45.8	0.039
0.00170	−0.003057	−0.0694	0.1757	−8.793	−40.8	0.042
0.00156	−0.005299	−0.0662	0.2141	−10.359	−42.4	0.045
0.00592	0.088317	−0.1428	0.3322	−12.425	−24.1	0.097
0.00583	0.108936	−0.1184	0.4182	−13.444	−20.3	0.153
0.00569	0.165765	−0.0565	0.4833	−13.049	−9.9	0.229
0.00804	0.228579	−0.0853	0.5537	−12.717	−10.6	0.320

The calorimetric measurements for the proposed compositions showed that the mixing enthalpy values reached a negative value regardless of the molar ratio used. Moreover, the values of the partial enthalpy of mixing of titanium presented in Table 2 show a step change in the values, corresponding to the transition from the homogeneous liquid region to the liquid–solid region. These values were marked with an asterisk symbol. Similar behavior was found in [19,20].

Table 4 presents the integral molar enthalpy of mixing data for respective sections in the vicinity of cross points of cross-sections (points 1–3 marked in Figure 3). The values of the integral molar mixing enthalpies were taken from Table 2 and Table 3. Taking into consideration the determined standard uncertainties (c.a. 0.8 kJ/mol), it can be said that the values of mixing enthalpy obtained for the two different cross-sections show good agreement.

The interaction parameters shown in Table 5 for the Ag–Mg and Mg–Ti systems were taken from the literature [5,14], while in the case of the Ag–Ti system, the parameters were developed taking into account the change in enthalpy of mixing from the measurement in this work (1 point for x_Ti_ = 0.0475) and based on the development of the interaction parameter taking into account the data from the interval for λ from 0 to −40 kJ/mol from Fitzner’s work [13] and for x = x_Ti_ 0 − 0.15.

(6) $$\lambda =\frac{\Delta_{\mathrm{mix}}H}{((1-x)\cdot x)}=10011.8-18866.8\cdot \left(1-2x\right)$$

The calculations included data for x_Ti_ = 0.15 from Fritzner’s work [13] because, according to Dezellus’ work [21], the liquidus for T = 1473 K is greater than this concentration (~0.175). Based on the presented above calorimetric data of the mixing enthalpy change for the Ag–Mg–Ti liquid solutions, the L123Liquidk parameters were calculated by the least squares method with the use of the proprietary optimization computer program (TerGexHm 1.0). The calculated standard deviation is equal to 0.432 kJ/mol, and the values of all the parameters in Equation (4) are shown in Table 5.

Applying the parameters from Table 5 and Equation (4), the integral and partial mixing enthalpies for the Ag–Mg–Ti liquid solutions were calculated for experimental cross-sections and are presented in Table 6.

**Figure 5:**
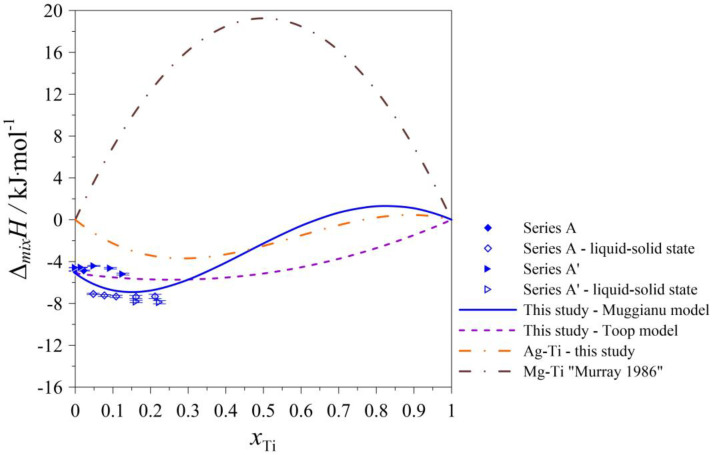
The integral mixing enthalpy of liquid (Ag_0.90_Mg_0.10_)_1−x_Ti_x_ alloys at 1294 K Series A and 1297 K Series A’ together with standard uncertainties [14].

**Figure 6:**
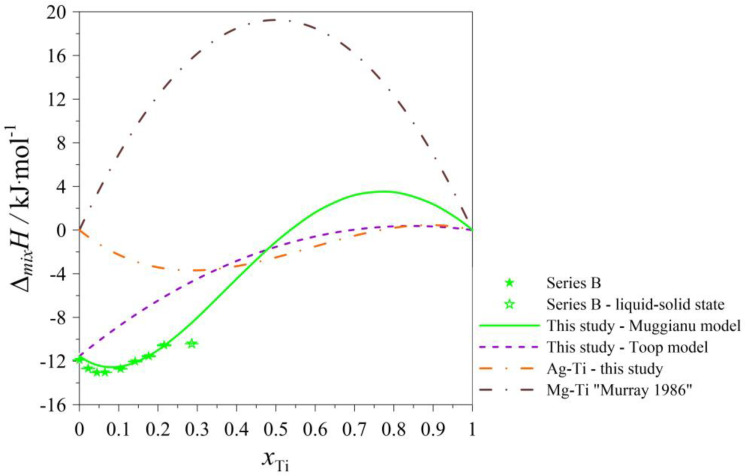
The integral mixing enthalpy of liquid (Ag_0.70_Mg_0.30_)_1−x_Ti_x_ alloys at 1294 K together with standard uncertainties [14].

**Figure 7:**
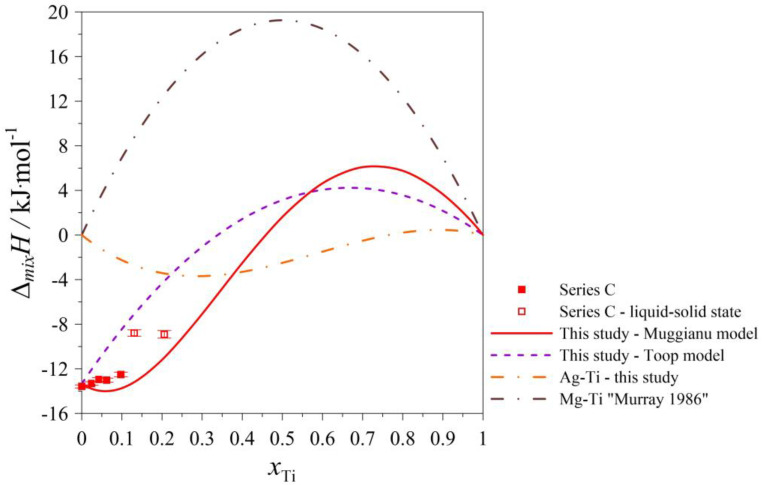
The integral mixing enthalpy of liquid (Ag_0.50_Mg_0.50_)_1−x_Ti_x_ alloys at 1294 K together with standard uncertainties [14].

**Figure 8:**
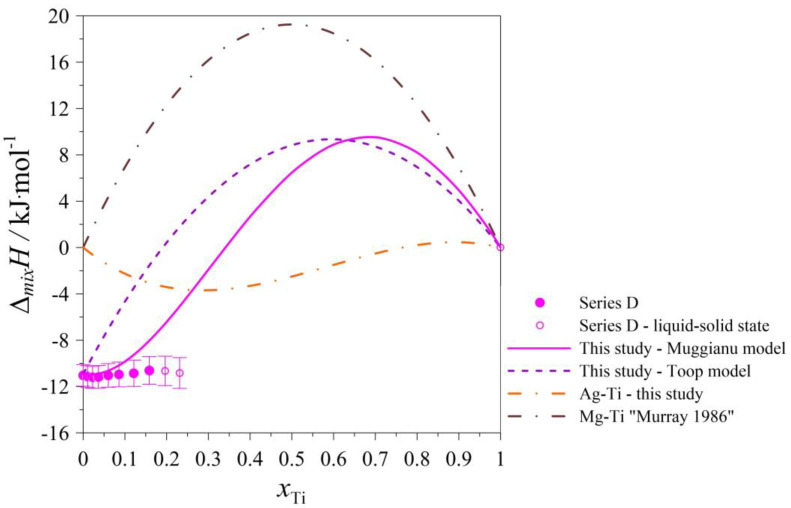
The integral mixing enthalpy of liquid (Ag_0.30_Mg_0.70_)_1−x_Ti_x_ alloys at 1294 K together with standard uncertainties [14].

**Figure 9:**
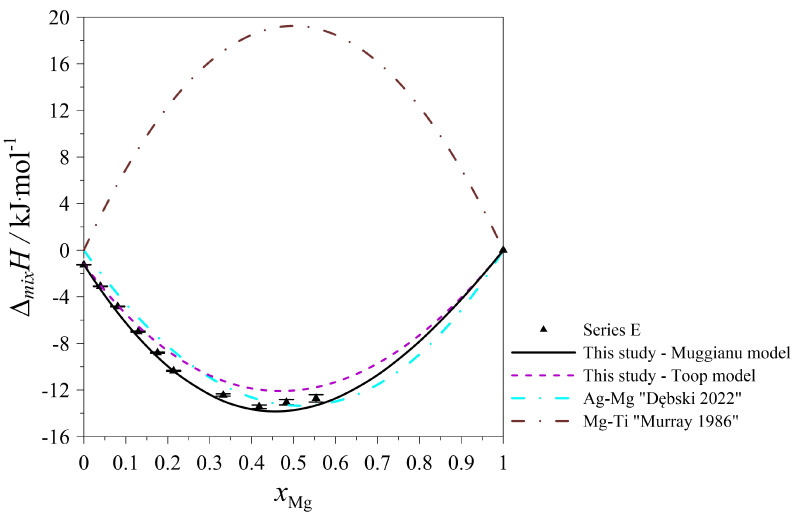
The integral mixing enthalpy of liquid (Ag_0.95_Ti_0.05_)_1−x_Mg_x_ at 1294 K together with standard uncertainties [5,14].

The determined values of the mixing enthalpy change for the Ag–Mg–Ti liquid solutions and those calculated with the use of Equation (4) (Muggianu model) based on the parameters presented in Table 5 are presented in Figure 5, Figure 6, Figure 7, Figure 8 and Figure 9, in which the solid lines show the calculated data of the mixing enthalpy change, and the symbols show the experimental values obtained by in this study. The dashed lines present data calculated with the use of Equation (5) (Toop’s model). The empty symbols indicate measurements in the liquid–solid state.

In Figure 5, Figure 6, Figure 7 and Figure 8, the trend of how the values of the calculated mixing enthalpies for the series A–D change is noticeable. Initially, negative values are reached. Further, simultaneously with an increase in the Ti content, an increase in enthalpy is observed—such a situation is observed at a certain concentration of Ti. After exceeding a maximal value of mixing enthalpy, a decrease in it is observed to 0 for *x*_Ti_ = 1. The maximal value of the mixing enthalpy for the A–D series is reached for different mole fractions of Ti. The curve describing the predicted enthalpy values using Toop’s model [17] also has a similar pattern. Only in the mole ratio x_Ag_/x_Mg_ = 9/1 case did the model predict negative values for the entire range. The largest difference between the minimum and maximum enthalpy is observed for the mole ratio x_Ag_/x_Mg_ = 1/1, which is 13,337 kJ/mol. Comparing the accuracy of the model to the results obtained experimentally, the best fit is seen for the mole ratio equaling x_Ag_/x_Mg_ = 7/3. The graph of the changes in enthalpy values for series E (Figure 9) has a completely different character compared to those showing the changes for series A–D. In this case, the curve has a parabolic shape, with a minimum at *x*_Mg_ = 0.5. This is similar to the curve obtained using Toop’s model. Over the entire range, the mixing enthalpy change reaches negative values, consistent with the experimental results shown in Table 3. Comparing the models used, it can be seen that in the case of alloys with variable Ag–Mg molar fraction ratios, and the Muggianu [16] model, the enthalpy reaches negative values, further reaches a maximum value greater than zero and at the end returns to zero again. The situation is different when analyzing the course of curves depicting Toop’s asymmetric model. Here, the integral mixing enthalpy change is slightly curvilinear. There are no anomalies similar to those in the symmetric model. Without information on the mixing enthalpy change for titanium-rich alloys, we cannot determine which model gives a better agreement with the experimental data. We are unable to check this by a calorimetric test due to the high vapor pressure of magnesium and the risk of damaging the calorimeter. In the case of the alloys with a mole ratio of Ag–Ti (Ag_0.95_Ti_0.05_), the agreement between the experimental and calculated (modeled) data for both models is the best.

## 4. Conclusions

These are the first conducted experimental studies of liquid Ag–Mg–Ti alloys and can be used in the future to optimize thermodynamic properties and phase diagram calculations. The results of the drop calorimetric measurements at 1294 K and 1297 K of the liquid Ag–Mg–Ti alloys show that the liquid solutions are characterized by negative deviations from ideal solutions. The minimum integral molar enthalpy value was −13.444 kJ/mol for alloy Ag_0.95_Ti_0.05_ and *x*_Mg_ = 0.4182.

The calorimetric data for the binary systems comprising the ternary system and the data from the calorimetric studies carried out in this investigation were used to develop a Muggianu model with ternary interaction parameters. The method of least squares was used, and the obtained standard deviation is equal to 0.432 kJ/mol. The data show that the value of the integral enthalpy of mixing for each of the selected cross-section points also reached negative values.

The modeling of the mixing enthalpy change of the ternary Ag–Mg–Ti liquid alloys based on the properties of binary solutions by Toop’s model was conducted. It was found that the best agreement between the modeled and experimental data is observed for the cross-sections of (Ag_0.95_Ti_0.05_)_1−x_Mg_x_ and (Ag_0.90_Mg_0.10_)_1−x_Ti_x_.

Further work on the Ag–Mg–Ti system is necessary to determine the structure of the alloys as well as their thermodynamic properties.

## Figures and Tables

**Table 1:** Specification of applied materials.

Chemical Name	Source	Purity [mass%]	Purification Method	Analysis Method
Magnesium	Goodfellow Cambridge Ltd., Huntingdon, England	99.9	None	Certified purity
Silver	Innovator Sp. z o.o., Gliwice, Poland	99.9	None	Certified purity
Titanium	Alfa Aesar, Thermo Scientific Kandel GmBH, Kandel, Germany	99.99	None	Certified purity
Argon	Pioniergas, Kraków, Poland	99.9999	None	Certified purity

**Table 4:** Integral molar enthalpy of mixing values near the points of intersection of experimental series.

Points of Intersection	A	A’	B	C	E
1	−4.855	−4.412			−4.828
2			−13.046		−12.425
3				−13.313	−13.049

**Table 5:** The binary and ternary interaction parameters applied for the calculation of the integral and partial mixing enthalpy change by Equation (4) for the Ag–Mg–Ti liquid alloys.

System	Interaction Parameters [J/mol]	Reference
Ag–Mg	LAg,MgLiquid0= −53,346.5	[5]
	LAg,MgLiquid1= −3694	
	LAg,MgLiquid2= −905.8	
Ag–Ti	LAg,TiLiquid0= −10,011.8	[This study]
	LAg,TiLiquid1= −18,866.8	
Mg–Ti	LMg,TiLiquid0= 77,020	[14]
Ag–Mg–Ti	LAg, Mg,TiLiquid1= −198,140.53	[This study]
	LAg, Mg,TiLiquid2= −434,984.54	
	LAg, Mg,TiLiquid3= 59,643.579	

**Table 6:** The partial and integral function of Ag–Mg–Ti liquid alloys.

*x_Ag_*	*x_Mg_*	*x_Ti_*	$\Delta \bar{H}$ *_Ag_*	$\Delta \bar{H}$ *_Mg_*	$\Delta \bar{H}$ *_Ti_*	∆_mix_*H*
			kJ/mol			
Series A and A’: (Ag_0.90_Mg_0.10_)_1−x_Ti_x_ alloys at T = 1297 K						
0.900	0.100	0.00	−0.661	−45.241	−31.065	−5.119
0.855	0.095	0.05	−0.846	−46.036	−21.246	−6.159
0.810	0.090	0.10	−1.601	−45.635	−13.281	−6.732
0.720	0.080	0.20	−4.214	−41.378	−2.077	−6.759
0.630	0.070	0.30	−7.397	−32.844	4.112	−5.726
0.540	0.060	0.40	−10.228	−20.588	6.663	−4.093
0.450	0.050	0.50	−11.967	−5.350	6.778	−2.264
0.360	0.040	0.60	−12.058	11.952	5.471	−0.580
0.270	0.030	0.70	−10.124	30.215	3.579	0.678
0.180	0.020	0.80	−5.971	48.155	1.753	1.290
0.090	0.010	0.90	0.412	64.306	0.464	1.097
0.000	0.000	1.00	8.855	77.020	0.000	0.000
Series B: (Ag_0.70_Mg_0.30_)_1−x_Ti_x_ alloys at T = 1297 K						
0.700	0.300	0.00	−5.504	−25.636	−37.763	−11.544
0.665	0.285	0.05	−5.750	−26.431	−21.224	−12.418
0.630	0.270	0.10	−7.018	−26.909	−8.348	−12.521
0.560	0.240	0.20	−11.373	−26.211	8,325	−10.994
0.490	0.210	0.30	−16.389	−22.449	15,769	−8.014
0.420	0.180	0.40	−20.381	−15.030	16.997	−4.467
0.350	0.150	0.50	−22.163	−3.854	14.526	−1.072
0.280	0.120	0.60	−21.044	10.680	10.377	1.615
0.210	0.090	0.70	−16.830	27.676	6.072	3.207
0.140	0.060	0.80	−9.826	45.740	2.637	3.479
0.070	0.030	0.90	−0.831	62.984	0.603	2.374
0.000	0.000	1.00	8.855	77.020	0.000	0.000
Series C: (Ag_0.50_Mg_0.50_)_1−x_Ti_x_ alloys at T = 1297 K						
0.500	0.500	0.00	−14.260	−12.413	−37.016	−13.337
0.475	0.475	0.05	−15.927	−11.731	−17.137	−13.994
0.450	0.450	0.10	−18.502	−11.589	−1.920	−13.733
0.400	0.400	0.20	−24.897	−11.623	17.046	−11.199
0.350	0.350	0.30	−30.906	−10.332	24.498	−7.084
0.300	0.300	0.40	−34.669	−6.209	24.377	−2.513
0.250	0.250	0.50	−35.001	1.576	19.947	1.617
0.200	0.200	0.60	−31.394	13.172	13.795	4.633
0.150	0.150	0.70	−24.019	28.056	7.831	6.087
0.100	0.100	0.80	−13.724	45.025	3.286	5.759
0.050	0.050	0.90	−2.032	62.198	0.717	3.653
0.000	0.000	1.00	8.855	77.020	0.000	0.000
Series D: (Ag_0.30_Mg_0.70_)_1−x_Ti_x_ alloys at T = 1297 K						
0.300	0.700	0.00	−26.360	−4.307	−16.539	−10.923
0.285	0.665	0.05	−32.359	−2.322	0.080	−10.762
0.270	0.630	0.10	−38.140	−1.256	12.461	−9.843
0.240	0.560	0.20	−48.008	−0.482	26.862	−6.419
0.210	0.490	0.30	−54.285	0.412	30.980	−1.904
0.180	0.420	0.40	−55.933	3.184	28.491	2.666
0.150	0.350	0.50	−52.554	8.951	22.431	6.465
0.120	0.280	0.60	−44.391	18.191	15.195	8.884
0.090	0.210	0.70	−32.325	30.742	8.539	9.524
0.060	0.140	0.80	−17.880	45.799	3.578	8.202
0.030	0.070	0.90	−3.219	61.919	0.787	4.946
0.000	0.000	1.00	8.855	77.020	0.000	0.000
Series E: (Ag_0.9525_Ti_0.0475_)_1−x_Mg_x_ alloys at T = 1297 K						
0.95250	0.00	0.04750	−0.142	−57.701	−22.948	−1.226
0.90488	0.05	0.04513	−0.338	−51.439	−22.404	−3.889
0.85725	0.10	0.04275	−0.836	−45.499	−22.547	−6.231
0.76200	0.20	0.03800	−2.760	−34.665	−24.180	−9.955
0.66675	0.30	0.03325	−5.915	−25.322	−26.385	−12.418
0.57150	0.40	0.02850	−10.246	−17.536	−27.643	−13.658
0.47625	0.50	0.02375	−15.640	−11.317	−26.376	−13.733
0.38100	0.60	0.01900	−21.925	−6.615	−20.952	−12.721
0.28575	0.70	0.01425	−28.873	−3.325	−9.678	−10.716
0.19050	0.80	0.00950	−36.200	−1.283	9.195	−7.835
0.09525	0.90	0.00475	−43.562	−0.267	37.473	−4.212
0.00000	1.00	0.00000	−50.559	0.000	77.020	0.000

## Data Availability

Raw data is available upon request.
